# Update on host-pathogen interactions in cystic fibrosis lung disease

**DOI:** 10.1186/s40348-016-0039-5

**Published:** 2016-02-23

**Authors:** Andreas Hector, Nina Frey, Dominik Hartl

**Affiliations:** Department of Pediatrics I, University of Tübingen, Tübingen, Germany

## Abstract

Bacterial and fungal infections are hallmarks of cystic fibrosis (CF) lung disease. In the era of long-term inhaled antibiotics and increasing CF patient survival, new “emerging” pathogens are detected in CF airways, yet their pathophysiological disease relevance remains largely controversial and incompletely defined. As a response to chronic microbial triggers, innate immune cells, particularly neutrophils, are continuously recruited into CF airways where they combat pathogens but also cause tissue injury through release of oxidants and proteases. The coordinated interplay between host immune cell activation and pathogens is essential for the outcome of CF lung disease. Here, we provide a concise overview and update on host-pathogen interactions in CF lung disease.

## Manuscript

### CF lung disease

Lung disease determines the morbidity and mortality of patients with cystic fibrosis (CF), a lethal monogenetic disease caused by mutations in the cystic fibrosis transmembrane conductance regulator (CFTR) gene [21]. Hallmarks of CF lung disease are its chronic, non-resolving and perpetuating nature. Importantly, a key characteristic of CF lung disease is the early and maintained interplay of microbial infection and severe airway inflammation [16]. The altered CF lung environment, characterized by mucus obstruction, airway surface liquid dehydration, pH dysregulation (lower, acidic pH), and high burden of extracellular proteases (mainly neutrophil elastase and matrix metalloproteases) favors microbial airway colonization and abnormal/inefficient host immunity [16, 21]. While inflammation in general is essential and indispensable to clear microbial infections and restore tissue integrity and homeostasis in acute infective conditions such as bacterial pneumonia, the pro-inflammatory response mechanisms activated in CF lung disease seem to be acting in an excessive, non-balanced, and therefore perpetuated manner [6]. The resulting immune cell infiltration leads to irreversible tissue remodeling with bronchiectasis and loss of lung function. Overall, host-pathogen interactions in CF are complex since they (1) depend on the individual CFTR mutation class, (2) evolve mutually in a temporal and spatial manner, and (3) are regulated by bacterial and fungal phenotypes, such as biofilm formations [17, 28, 35]. Here, we aim to provide a concise overview on host-pathogen interactions in CF lung disease in order to shed light on new avenues for future research and treatment approaches.

### Microbial airway colonization in CF lung disease

CF airways are mainly colonized by specific bacteria and fungi [28]. Among bacteria, *Pseudomonas aeruginosa* and *Staphylococcus aureus* are the most abundant and consequently most thoroughly studied pathogens. In early infancy, CF airways are typically colonized with *S. aureus* and *Haemophilus influenzae*. Later on in childhood, *P. aeruginosa* predominates and modulates disease outcome substantially [3]. Upon chronic colonization, *P. aeruginosa* can adapt this phenotype by conversion into a mucoid form that is more resistant to antibiotics and host defense. The majority of inhaled or systemic antibiotics used to treat CF patients is actually directed against *P. aeruginosa* in order to eradicate or suppress this opportunistic Gram-negative bacterium [7]. The underlying host-pathogen interaction mechanisms regulating the CF-characteristic microbial “switch” from *S. aureus* and *H. influenzae* to *P. aeruginosa* remain, however, controversial and incompletely understood, but probably involve pathogen-derived factors, such as pyocyanin and host-derived immune factors as well as environmental influences. In the era of commonly and early used inhaled antibiotics and prolonged patient survival, new “emerging” pathogens are increasingly detected in CF airway fluids, particularly fungi, such as *Aspergillus fumigatus* [2, 26], *Candida albicans* [9, 10], and *Scedosporium species* [22], and the bacteria *Stenotrophomonas maltophilia* [13], *Achromobacter xylosoxidans* [12, 15], methicillin-resistant *S. aureus* (MRSA) [14], *Burkholderia cepacia* [11, 25], and atypical mycobacteria (nontuberculous mycobacteria, NTMs) [1, 23], which are often hard to treat in the clinics due to antibiotic resistance patterns [7, 8, 31, 33, 34]. Whereas the prevalence (or at least the detection rate) of these microbial species increases in most CF centers, their pathophysiological disease relevance for the outcome of CF lung disease remains controversial and poorly defined. MRSA [14], *B. cepacia*, and NTMs [23] are broadly accepted as harmful CF pathogens, while for other rare species (such as *S. maltophilia* [13, 30, 32]), this is less clear.

### Host immunity

Faced with the presence of bacterial and fungal microbial species, the host immune response reacts by recruiting innate and adaptive immune cells into the infected CF airway compartment. Among innate immune cells, neutrophils are the most rapid and predominant cell type transmigrating into CF airways, while in adaptive immunity, T-helper cell type 2 (Th2) and Th17 cell responses are predominant [16], while regulatory T cell responses are impaired [18]. Remarkably, phagocytic innate immune cells (neutrophils and macrophages) preferentially accumulate within the airway compartment, whereas, in contrast, lymphocytes are mainly found in lung tissues, but are very low within the airway lumen [24]. The underlying migratory and/or tissue homeostatic mechanisms regulating this distinct immune cell tissue compartment localization/distribution remain to be defined, yet recent studies suggest that neutrophils can suppress and thereby dampen T cell activity at sites of inflammation [19]. When innate immune cells are in physical contact with pathogens, several factors decide which anti-microbial defense mechanisms are employed; phagocytotic uptake is the most rapid and principal effector function against smaller bacteria and fungi, particularly after antibody- and/or complement-mediated opsonisation [20]. If pathogen size exceeds a critical threshold or pathogens shield themselves through biofilms, neutrophils are unable to efficiently phagocytose pathogens and utilize their extracellular host defense armamentarium, consisting of neutrophil extracellular trap (NET) formation [4] and the release of intracellularly stored anti-microbial effector proteins (such as defensins and proteases) [20]. In CF airways in vivo, probably a combination of these host defense mechanisms is operative, yet studies comparing the relative contribution of these distinct neutrophil functionalities to host defense outcome within the CF airways are lacking (to the best of our knowledge). Beyond cellular mechanisms, studies involved a dysregulated ceramide homeostasis/turnover in CF lung disease by showing that ceramide accumulates in CF airways and mediates inflammation, cell death, and infection susceptibility [29].

## Conclusion

Our understanding of host-pathogen interactions in CF lung disease is continuously and substantially renewed by current findings in microbiology (for instance by the microbiome [27]) and immunology/cell biology (for instance by the discovery of NET formation [5]). The challenge for the future remains to combine insights from these two often disconnected scientific fields and to translate them efficiently into the complex pathogenesis of CF lung disease. A more precise dissection/stratification of host-pathogen interaction components into beneficial (anti-infective, host defensive) and harmful (collateral tissue damage) subtypes could pave the way to develop novel optimized strategies for biomarker development and therapeutic drug targeting in CF lung disease.
